# Supplementary material for: Efficient Micropropagation of Sedum sediforme and S. album for Large-Scale Propagation and Integration into Green Roof Systems
Source: Plants (Basel). 2025 Jun 13;14(12):1819. doi: 10.3390/plants14121819 (PMC12196765; doi:10.3390/plants14121819)
Supplement: Supplementary file 1 [file plants-14-01819-s001.zip › plants-3660056-supplementary.pdf]

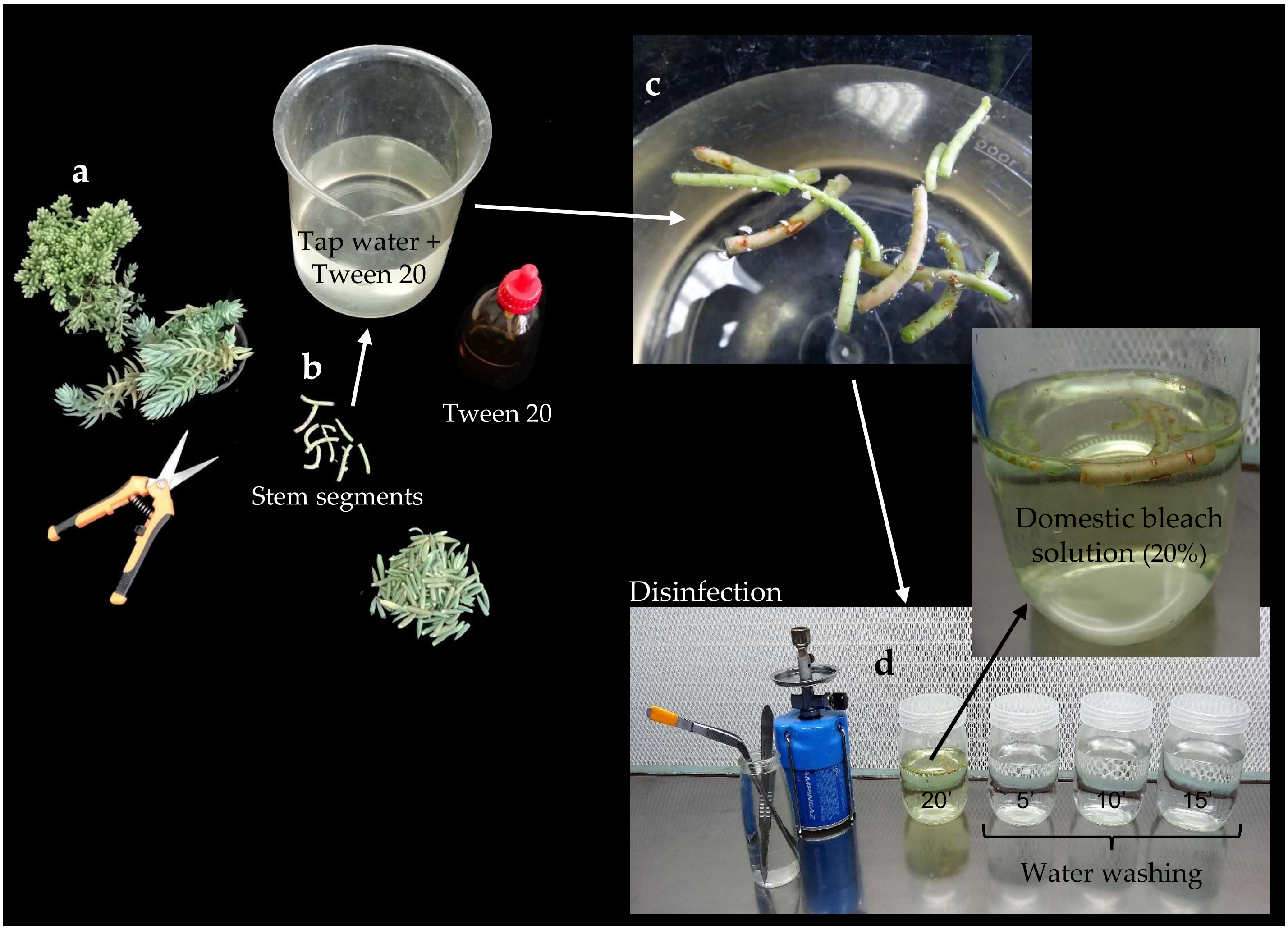

Figure S1. Disinfection of *S. sediforme* and *S. album*. After harvesting the upper stem segments of *S. sediforme* and *S. album* (a), all leaves were removed (b). Stem segments without leaves were carefully washed with tap water and Tween20 surfactant (c). Finally, the stem segments are disinfected with household bleach supplemented with Tween20 (d).

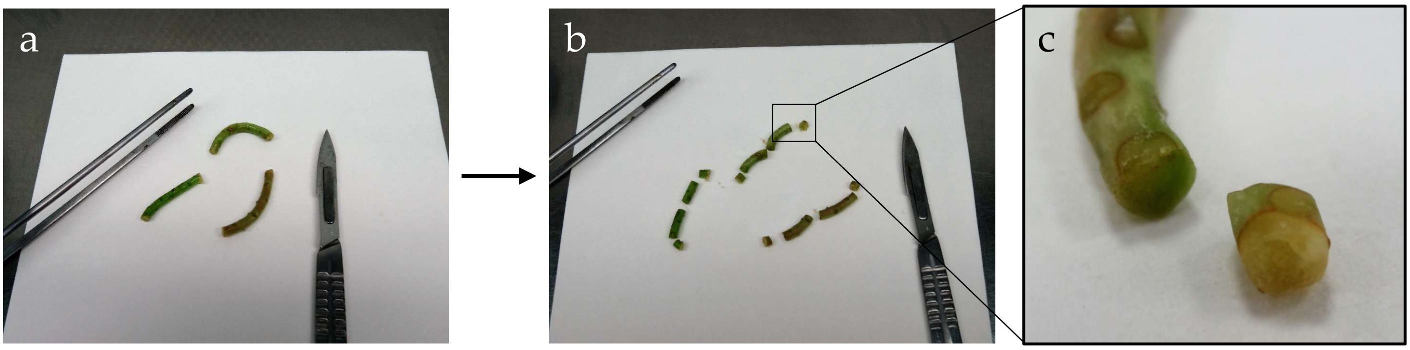

Figure S2. Obtaining nodal explants. Stem segments (a) were cut into smaller nodal explants (b) containing at least one axillary bud. The ends of the stem segments, impaired by treatment with the disinfectant solution, were removed (c).

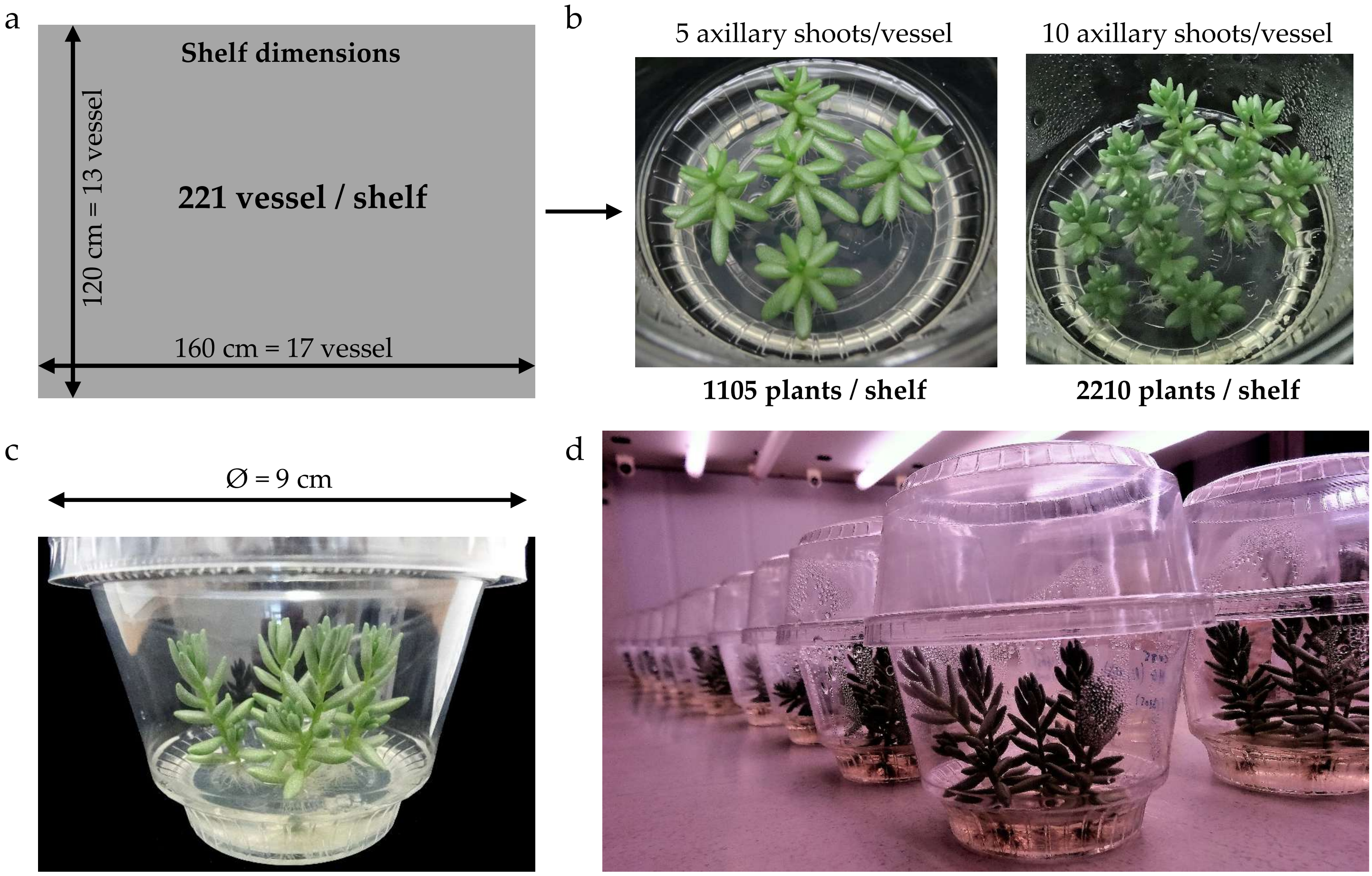

Figure S3. Micropropagation potential of *Sedum* under *in vitro* culture conditions. Dimensions of a shelf in the plant growth chamber of our laboratory. (a). Cultivation of 5 or 10 axenic plants in a vessel (b). Diameter of a vessel (c). *In vitro* growth of *S. album* on one of the shelves of our plant culture chamber (d).
